# Length of metacarpal and metatarsal bones in five Iranian sheep breeds and their associations with ungula measurements

**DOI:** 10.1186/s12917-021-03076-5

**Published:** 2021-12-06

**Authors:** Samaneh Azarpajouh, María Pia Munita, Julia Adriana Calderón Díaz

**Affiliations:** 1 Department of Clinical Science, Shahrekord College of Veterinary Medicine, Shahrekord, Iran; 2 Independent Researcher, Statesboro, GA 30461 USA; 3 Veterinary Pathobiology, School of Veterinary Medicine, University College Dublin, Belfield, Dublin 4, Ireland; 4 Pig Development Department, Animal and Grassland Research and Innovation Centre, Teagasc, Moorepark, Fermoy, Co Cork Ireland

**Keywords:** Ewe, Morphometry, Sheep, Ungula measurements, Ungula function

## Abstract

**Background:**

This study aimed to measure the length of metacarpal and metatarsal bones in five Iranian sheep breeds and to correlate the length of the bones with ungula measurements. Thoracic and pelvic limbs of 2-year-old, previously untrimmed, pastured Afshari, Moghani, Kurdi, Makoui, and Lori–Bakhtiari ewes, (*n* = 20 ewes per breed) were collected after slaughter. The following lengths were recorded in the metacarpal and metatarsal bones: from the margo proximalis lateralis to the lateral (L1) and medial (D1) cartilago physialis; from the margo proximalis lateralis to the margo abaxialis of the lateral (L2) and medial (D2) caput; from the cartilago physialis lateralis to the margo abaxialis of the lateral caput (X1); from the cartilago physialis medialis to the margo distalis of the caput ridge (X2) and from the margo axialis of cartilago physialis to the margo axialis of the lateral caput (X3). Additionally, measurements of the ungula including pars dorsalis length, pars mobilis lateralis and medialis height, pars dorsalis height to the ground and to the solea cornea, thickness of the solea in the pars dorsalis, pars mobilis lateralis and medialis, solea cornea length and angulus dorsalis were recorded in the medial and lateral digits of the thoracic and pelvic limbs. Data on length of the metatarsal and metacarpal bones were analysed using mixed model equations while Pearson correlations were calculated between metacarpal and metatarsal bones and ungula measurements.

**Results:**

Lori- Bakhtiari and Moghani ewes had greater L1, L2, and D1 and D2 while X1, X2 and X3 was greater in Kurdi ewes (*P* < 0.05). Measurements such as L1, L2, D1 and D2 were greater in the metatarsal than in metacarpal bones (*P* < 0.05) and the opposite was observed for X1, X2 and X3 (*P* < 0.05). No asymmetry was observed between the lateral and medial measurements (*P* > 0.05). Low to moderate correlations were observed between bone and ungula measurements (*P* < 0.05).

**Conclusion:**

Under the conditions of this study, differences in metacarpal and metatarsal bone measurements were observed between breeds but no asymmetry was observed between lateral and medial bones. Results indicate an association between metacarpal and metatarsal bones ungula measurements. This could provide baseline information for the development and/or improvement of current ungula health protocols in the studied sheep breeds.

**Supplementary Information:**

The online version contains supplementary material available at 10.1186/s12917-021-03076-5.

## Background

Sheep (*Ovis aries*) are multi-purpose animals, reared worldwide predominantly for their meat, milk and wool production. Versatility and adaptability are characteristics that typify *Ovis aries* thus, they are present in every continent and landscape, serving different functions and reach a population of more than 1.2 billion heads worldwide [1]. Flock health plays an important role in sheep production performance [2, 3]. In sheep, lameness is a significant problem for animal welfare [4, 5] affecting sheep health and productivity [6]. Although lameness is a multifactorial condition [7, 8], lesions in the ungula [9] and limb conformation [10] are recognised as some of its main causes.

Functional trimming is one of the most widely used approaches to maintain health of the ungula by providing appropriate weight distributions between the lateral and medial digits. However, despite regular trimming, the lateral digit usually returns to bear markedly more weight than the medial digit after a short time [11]. The reasons for this are unknown but it has been hypothesised that it could be due to a difference in length of the lateral and medial metacarpal and metatarsal capita. Previous studies reported that lateral capita were longer than medial capita in cattle [12–14] and buffaloes [15, 16] with indications of noticeable differences in metacarpal and metatarsal bone characteristics between breeds [16, 17]. Additionally, positive correlations are reported between the length of the capita and length and width of the ungula in cows and calves [12, 18] suggesting that these anatomical differences could explain, to some extent, the higher predisposition of the lateral digit to diseases [14].

To date, studies informing about differences in the length of metacarpal and metatarsal bones in sheep are scarce and there is no information about their associations with features of the ungula in sheep. This could have implications for designing ungula health protocols for sheep considering these characteristics. Thus, the objectives of this study were 1) to measure the length of metacarpal and metatarsal bones in five Iranian sheep breeds, namely Lori-Bakhtiari, Moghani, Makoui and Afshari and Kurdi and, 2) to investigate correlations between the length of metacarpal and metatarsal bones with ungula measurements.

## Results

**Fig. 1:**
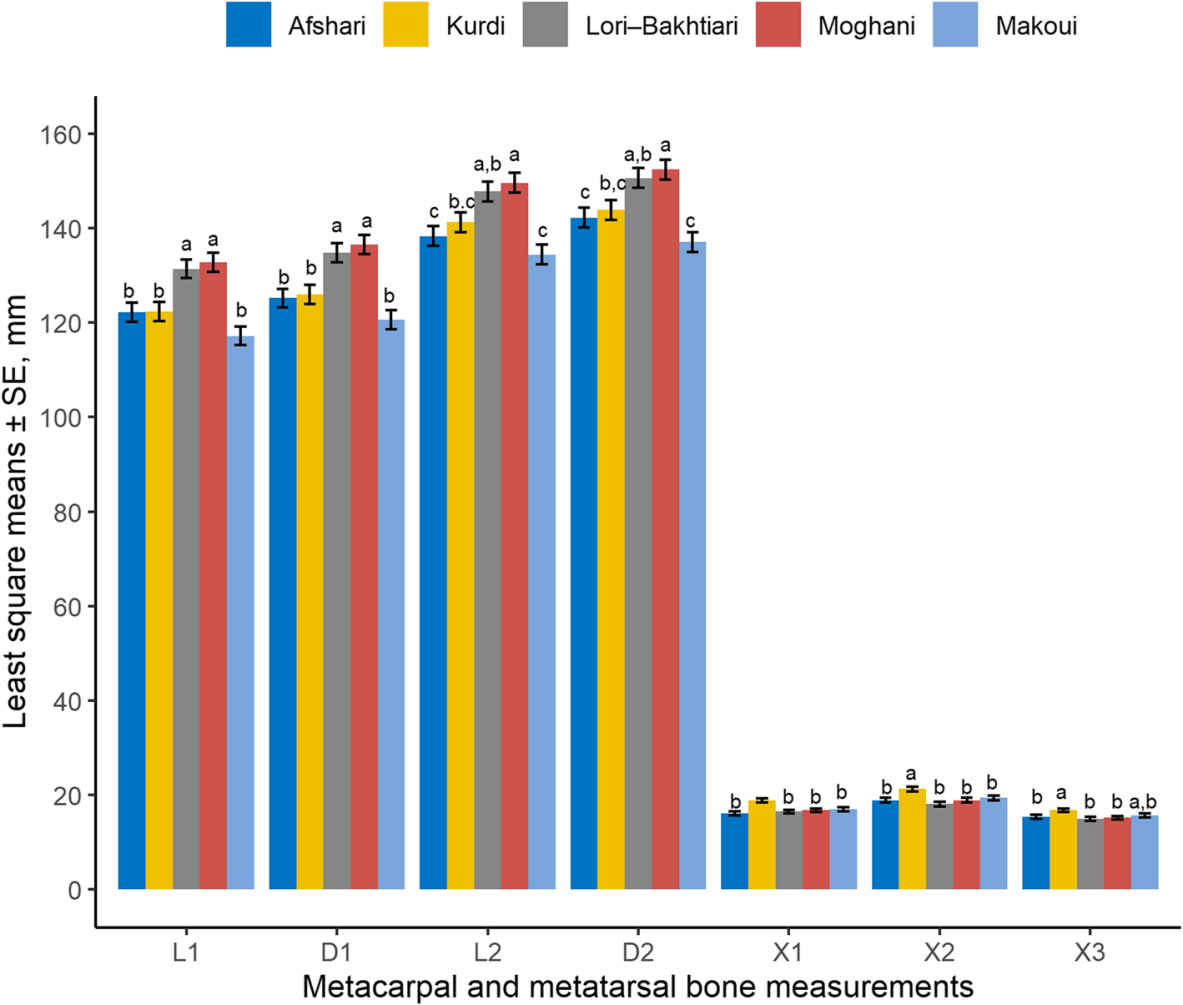
Differences [least square means ± standard error (SE)] in the distance from the margo proximalis lateralis to the lateral (L1) and medial (D1) cartilago physialis; distance from the margo proximalis lateralis to the margo abaxialis of the lateral (L2) and medial (D2) caput; distance from cartilago physialis lateralis to the margo abaxialis of the lateral caput (X1); distance from the cartilago physialis medialis to the margo distalis of the caput ridge (X2); and distance from the margo axialis of the cartilago physialis axialis to the margo axialis of the lateral caput (X3) measured in 2-year-old untrimmed pastured ewes from five Iranian sheep breeds. ^a-c^ Significant differences (*P* < 0.05) between breeds

**Fig. 2:**
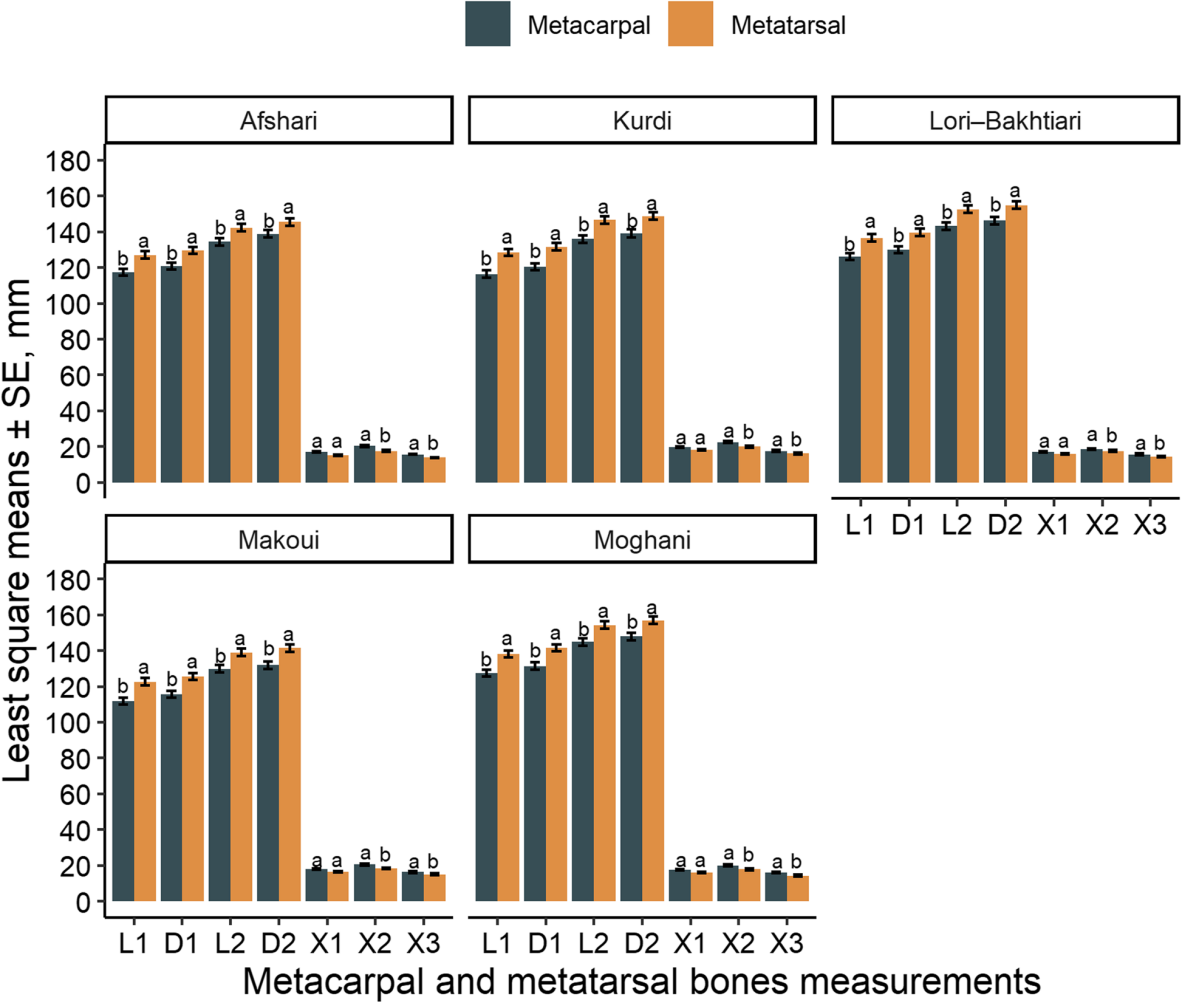
Differences [least square means ± standard error (SE)] between metacarpal and metatarsal distance from the margo proximalis lateralis to the lateral (L1) and medial (D1) cartilago physialis; distance from the margo proximalis lateralis to the margo abaxialis of the lateral (L2) and medial (D2) caput; distance from cartilago physialis lateralis to the margo abaxialis of the lateral caput (X1); distance from the cartilago physialis medialis to the margo distalis of the caput ridge (X2); and distance from the margo axialis of the cartilago physialis axialis to the margo axialis of the lateral caput (X3) in 2-year-old untrimmed pastured ewes from five different Iranian sheep. ^a-b^ Significant differences (*P* < 0.05)

Distance from the margo proximalis of the metapodial bone to the cartilago physialis lateralis (L1) and medialis (D1) was larger in Lori-Bakhtiari and Moghani ewes compared with all other breeds (*P* < 0.001). Distance from the margo proximalis lateralis of the metapodial bone to the margo abaxialis of the capita lateralis (L2) and medialis (D2) was larger for Moghani ewes and shorter for Makoui and Afshari ewes (*P* < 0.001; Fig. 1). Distance from the cartilago physialis lateralis to the margo abaxialis of the caput lateralis (X1), distance from the cartilago physialis to the margo distalis of the caput ridge (X2) and distance from the cartilago physialis axialis to the margo axialis of the lateral caput (X3) was larger in Kurdi ewes (*P* < 0.001) compared with the other breeds (Fig. 1). Furthermore, for all breeds L1, D1, L2 and D2 were larger in the metatarsal bones compared with the metacarpal bones (*P* < 0.05; Fig. 2) while X1, X2 and X3 were larger in the metacarpal capita compared with the metatarsal capita (*P* < 0.05). Differences between lateral and medial measurements were only observed for L2 in Afshari sheep (*P* < 0.05) with the medial distance being 2 mm longer than the lateral side. A similar tendency was observed for longer medial L1 (*P =* 0.07) and D1 (*P =* 0.08) than lateral measurements in the same breed (Table 1).

**Table 1:** Differences [least square means ± standard error (SE)] between lateral and medial measurements for the length of metacarpal and metatarsal bones in 2-year-old untrimmed pastured ewes from five different Iranian sheep breeds

Trait	Afshari			Kurdi			Lori-Bakhtiari			Moghani			Makoui		
	Lateral	Medial	SE	Lateral	Medial	SE	Lateral	Medial	SE	Lateral	Medial	SE	Lateral	Medial	SE
**L1**^**1**^**, mm**	121.5^(b)^	122.8^a^	2.00	122.5^a^	122.4^a^	2.00	1314^a^	131.3^a^	2.00	133.0^a^	132.7^a^	2.00	1173^a^	117.2^a^	2.00
**L2**^**2**^**, mm**	137.5^b^	139.1^a^	2.10	141.3^a^	141.3^a^	2.10	147.8^a^	147.8^a^	2.10	149.7^a^	149.5^a^	2.10	134.4^a^	134.4^a^	2.10
**D1**^**3**^**, mm**	124.4^(b)^	126.0^b^	2.00	125.5^a^	126.5^a^	2.00	134.7^a^	134.9^a^	2.00	136.4^a^	136.6^a^	2.00	120.4^a^	120.9^a^	2.00
**D2**^**4**^**, mm**	141.8^a^	142.5^a^	2.10	143.5^a^	144.4^a^	2.10	150.4^a^	150.8^a^	2.10	152.4^a^	152.4^a^	2.10	136.9^a^	13.47^a^	2.10
**X1**^**5**^**, mm**	15.96^a^	16.3^a^	0.40	18.9^a^	18.9^a^	0.40	16.4^a^	16.6^a^	0.40	16.8^a^	16.9^a^	0.40	17.1^a^	17.2^a^	0.40
**X2**^**6**^**, mm**	19.0^a^	18.9^a^	0.50	21.5^a^	21.1^a^	0.50	18.0^a^	18.2^a^	0.50	18.9^a^	18.8^a^	0.50	19.4^a^	19.4^a^	0.50
**X3**^**7**^**, mm**	14.7^a^	14.9^a^	0.30	16.9^a^	16.7^a^	0.50	15.0^a^	151^a^	0.50	15.2^a^	15.2^a^	0.50	15.7^a^	15.6^a^	0.50

^a-b^ *P* < 0.05

^(a-b)^ *P* < 0.10

^1^ Distance from the margo proximalis lateralis to the lateral cartilago physialis

^2^ Distance from the margo proximalis lateralis to the margo abaxialis of the lateral caput

^3^ Distance from the margo proximalis lateralis to medial cartilago physialis

^4^ Distance from the margo proximalis lateralis to the margo abaxialis of the medial caput

^5^ Distance from cartilago physialis lateralis to the margo abaxialis of the lateral caput

^6^ Distance from the cartilago physialis medialis to the margo distalis of the caput ridge

^7^ Distance from the margo axialis of the cartilago physialis axialis to the margo axialis of the lateral caput

Differences in ungula measurements between breeds, limbs (i.e. thoracic and pelvic) and digits (i.e. lateral and medial) were previously reported by Azarpajouh et al. [19]. Briefly, the authors reported that Afshari and Makoui sheep had lower values for all ungula measurements. Additionally, for all breeds, differences were observed between thoracic and pelvic limbs but not between lateral and medial digits. Descriptive statistics for ungula measurements are presented in Supplementary Fig. 1.

Pearson’s correlations between caput length and ungula measurements are presented in Table 2. Positive moderate correlations (*P* < 0.05) were observed between the distance from the margo proximalis of the metapodial bone to the cartilago physialis lateralis and medialis and between the abaxial border of the caput lateralis and medialis and pars dorsalis length, pars dorsalis length to the corona, pars dorsalis height to the ground and to the solea cornea, length of the solea cornea, pars dorsalis height to the solea cornea, and pars mobilis lateralis and medialis height ratio in the ungula. Additionally, there was a low negative correlation between pars mobilis lateralis and medialis height and the distance from the margo proximalis lateralis of the metapodial bone to the lateral border of the cartilago physialis. Distance from the cartilago physialis lateralis to the margo abaxialis of the lateral caput was positively correlated with pars dorsalis length, pars dorsalis height to the ground, solea cornea length, and solea cornea height in the ungula, but it was negatively correlated with pars dorsalis height to the solea cornea to pars mobilis lateralis and medialis height ratio (*P* < 0.05). There were positive correlations between the distance from the cartilago physialis to the distal end of the caput ridge and pars dorsalis length, pars dorsalis height to the ground and pars mobilis lateralis and medialis height in the ungula. Negative correlations were observed between the distance from the cartilago physialis to the caput distalis ridge and pars dorsalis length to the corona and pars dorsalis height to the solea cornea to pars mobilis lateralis and medialis height ratio in the ungula (*P* < 0.05). Finally, distance from the margo axialis of the cartilago physialis to the margo axialis of the caput lateralis had a low positive correlation with pars mobilis lateralis and medialis height and a low negative correlation with pars dorsalis height to the solea cornea to pars mobilis lateralis and medialis height ratio (*P* < 0.05).

**Table 2:** Pearson’s correlations between metacarpal and metatarsal bones and hoof size measurements^a^ in 2-year-old untrimmed pastured ewes

	L1, mm	D1, mm	L2, mm	D2, mm	X1, mm	X2, mm	X3, mm
**Pars dorsalis length, mm**	0.29^*^	0.30^*^	0.33^*^	0.31^*^	0.16^*^	0.10^*^	0.07
**Pars dorsalis length to the corona, mm**	0.32^*^	0.33^*^	0.31^*^	0.31^*^	−0.07	− 0.14^*^	− 0.07
**Pars dorsalis height to the ground, mm**	0.30^*^	0.33^*^	0.35^*^	0.34^*^	0.24^*^	0.17^*^	0.07
**Pars dorsalis height to the solea cornea, mm**	0.34^*^	0.36^*^	0.36^*^	0.36^*^	0.09	0.01	0.01
**Solea length, mm**	0.31^*^	0.33	0.34^*^	0.34^*^	0.14^*^	0.06	0.04
**Solea cornea in the pars dorsalis region, mm**	0.07	0.08	0.08	0.09	0.08	0.03	−0.02
**Solea cornea thickness in the pars mobilis lateralis/medialis region, mm**	−0.03	− 0.02	−0.03	− 0.03	0.00	0.02	0.01
**Pars mobilis lateralis and medialis height, mm**	−0.10^*^	−0.07	− 0.03	−0.02	0.30^*^	0.31^*^	0.12^*^
**Pars dorsalis angle, degree**	−0.01	0.00	−0.03	−0.01	− 0.08	−0.08	− 0.05
**Pars dorsalis height to the solea cornea to pars mobilis lateralis and medialis height ratio**	0.32^*^	0.31^*^	0.28^*^	0.27^*^	−0.23^*^	−0.29^*^	− 0.11^*^

^a^Metacarpal and metatarsal bone measurements include: Distance from the margo proximalis lateralis to the lateral (L1) and medial (D1) cartilago physialis; distance from the margo proximalis lateralis to the margo abaxialis of the lateral (L2) and medial (D2) caput; distance from cartilago physialis lateralis to the margo abaxialis of the lateral caput (X1); distance from the cartilago physialis medialis to the margo distalis of the caput ridge (X2); and distance from the margo axialis of the cartilago physialis axialis to the margo axialis of the lateral caput (X3)

^*^P < 0.05

## Discussion

Sheep are highly adaptable ruminants, they can be raised in a broad variety of agricultural systems and have the potential of generating multiple products. Extensive pasture based flocks usually count with minimal human intervention, therefore adaptability and survival traits are of major importance for a more self-sufficient animal lifestyle [20]. Fat-tailed sheep are distinguished by their fat deposition in the tail, they are also recognised for their ability to handle extreme environmental conditions and cope well with migration and winter [21]. Lameness is one of the most important animal welfare and economic challenges across the sheep sector [22]; however, anatomical variations have not been a source of study to explain the major causes of lameness. This study aimed to measure the length of the metacarpal and metatarsal bones and to investigate the relations between these measurements and features of the ungula in five Iranian sheep breeds in an effort to provide useful information to improve preventive ungula health programs. To our knowledge, this is the first time that these measurements have been reported for fat-tail multipurpose sheep breeds.

Differences were observed between the five sheep breeds for all bone measurements which is similar to results previously reported in cattle after slaughter [16, 23]. These differences are likely due to the size/weight of the animals as bigger/ heavier breeds such as Kurdi and Lori-Bakhtiari generally had longer bones than smaller/lighter breeds. These findings are in concordance with those reported by Silva el at [16] of a positive association between bone length and body weight in cattle. However, as body weight was not recorded in the present study, further investigation is required regarding the associations between metapodial bones measurements and body weight in sheep.

Nacambo et al. [12] and Nourinezhad et al. [15] reported differences in length between metatarsal and metacarpal bones in cattle and buffalos, respectively. This characteristic was also observed in the present study, and likewise, the metatarsal bones were longer than metacarpal bones, a characteristic shared by all studied breeds. It has been hypothesised that such differences could be related to the angulation of the lower part of the pelvic limbs in ungulates, which requires longer metatarsal bones for correct gait and weigh bearing [12, 24]. On the contrary, metacarpal capita were longer than metatarsal capita although differences were small (1 to 2 mm) and may not be biologically relevant. However, this difference was observed across all breeds, and it could potentially be explained by locomotor kinematics and by the mechanical environment in which the development of the joint surfaces took place [25]. Metatarsal and metacarpal bones are major parts of metacarpo and metatarso-phalangeae joints and variables such as mechanical load and floor surface could influence the growth of such bones. Metatarsal caput bear less weight than their metacarpal counterparts as they do not handle the thorax and head weight and thus, the greater support demand in the thoracic limbs might require larger joint surface within the joint.

Under the conditions of this study, we did not observe asymmetry between the lateral and medial side of bone and caput measurements in any of the sheep breeds except for Afshari sheep, where a tendency was observed. Our results are contrary of previous reports in cattle having longer lateral caput and metapodial bones [12] but similar to the findings reported by Nourinezhad et al. [16, 26] in water buffalos. Symmetric bones could contribute to distribute weight equally between the lateral and medial phalanges and ungula while walking or standing and thus, ultimately contributing to better health of the ungula by reducing the likelihood of developing lesions in the ungula. However, further investigations in the subject are required as there is no report about this relationship or about the prevalence of lesions in the ungula in Iranian sheep breeds.

The symmetry in bone and caput length could explain, at least in part, the symmetry observed in ungula measurements such as pars dorsalis length in the same sheep breeds reported by Azarpajouh et al. [19], suggesting that caput and ungula measurements in sheep are indeed associated. This association was confirmed when examining the correlations between bone and caput length and ungula measurements in this study. An advantage of this study is that sheep were not previously trimmed allowing to investigate *undisturbed* associations. Although most correlations were moderate, they indicate that animals with longer bone and caput lengths would also have, for example, longer pars dorsalis which is one of the ungula characteristics altered by functional trimming since overgrowth of the ungula predisposes the limb to mild to moderate lameness [27]. By knowing this, functional ungula trimming protocols can include a baseline knowledge about which sheep breed have naturally longer pars dorsalis versus those suffering from pars dorsalis overgrowth and thus, functional trimming protocols could be improved accordingly.

A limitation of this study is that we only used a small sample size and only included females, and thus, measurements derived from this study are not representative of all five Iranian sheep breeds in general. However, the measurements were recorded under standardised conditions and thus, comparisons between breeds are justified.

## Conclusion

Metapodial bones and caput length varied between sheep breeds with bigger/heavier breeds displaying longer lengths for all studied traits suggesting a possible association with body weight; however, this warrants further investigation. Although metatarsal bones were longer than metacarpal bones, no difference was observed between lateral and medial bones indicating that metapodial bone and caput asymmetry is absent in the studied breeds. Nonetheless, results indicate that metapodial bones measurements are associated with ungula growth patterns as they were moderately correlated. Results from this study could provide baseline information for the development and/or improvement of current ungula health protocols in the studied sheep breeds.

## Methods

### Sample population

Thoracic and pelvic limbs of 2-year-old, previously untrimmed, pastured ewes (*n* = 100 ewes) were collected after slaughter in 2007 within a 6-month period. Ewes came from five different Iranian sheep breeds: Afshari, Moghani, Kurdi, Makoui, and Lori–Bakhtiari. Twenty ewes were randomly selected from each breed. Ewes were reared in a mountainous area in a western region of Iran. Based on farm records, ewes were never trimmed or lame and had not suffered from laminitis.

### Measurements

**Fig. 3:**
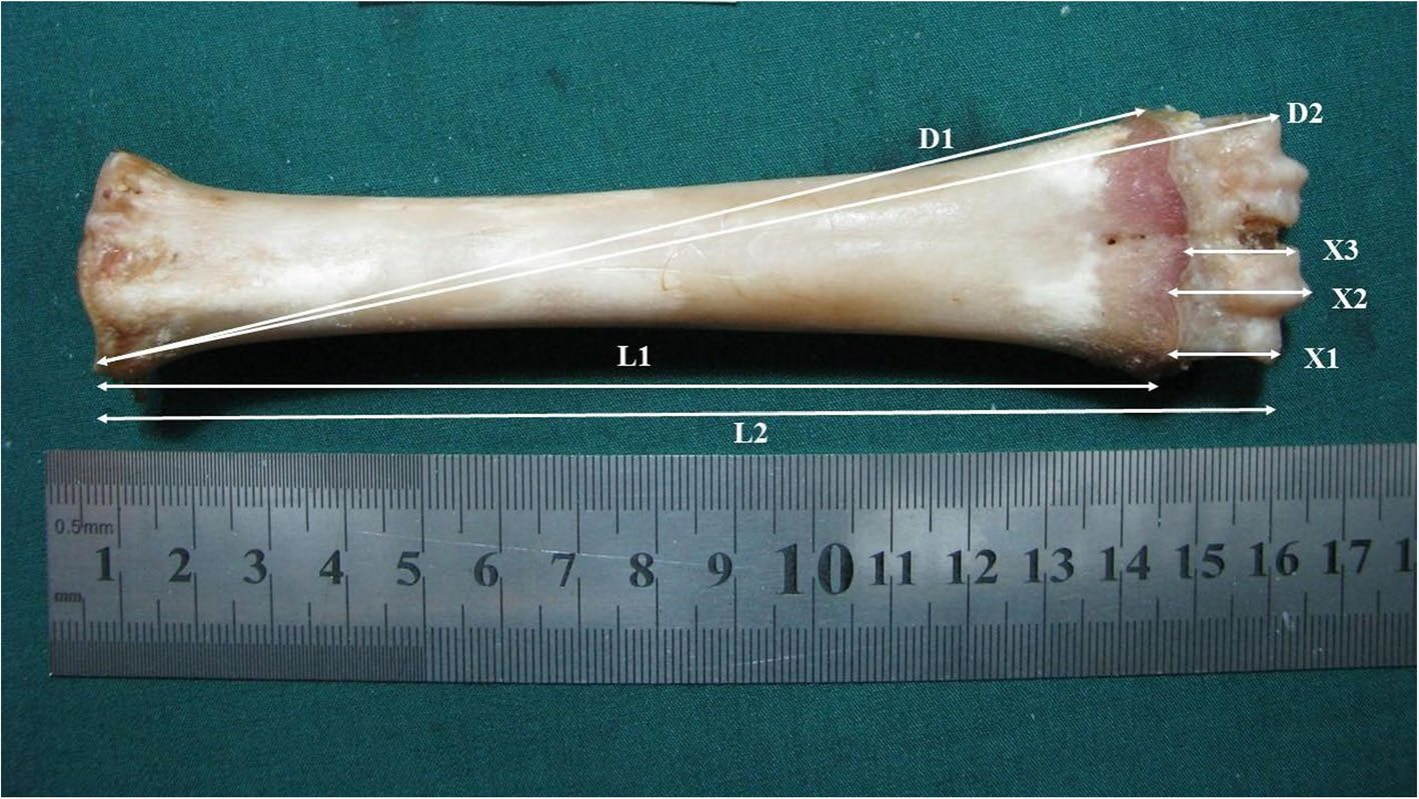
Topographical representation of different metacarpal and metatarsal bone measurement of 2-year-old untrimmed pastured ewes from five different Iranian sheep breeds. **L1)** distance from the margo proximalis lateralis to the lateral cartilago physialis; **D1)** distance from margo proximalis lateralis to the medial cartilago physialis; **L2)** distance from the margo proximalis lateralis to the margo abaxialis of the lateral caput; **D2)** distance from the margo proximalis lateralis to the margo abaxialis of the medial caput; **X1)** distance from the cartilago physialis lateralis to the margo abaxialis of the lateral caput; X2) distance from the cartilago physialis medialis to the margo distalis of the caput ridge; and **X3)** distance from the margo axialis of the cartilago physialis axialis to the margo axialis of the lateral caput

Thoracic and pelvic limbs were removed from above the carpal and tarsal joints. Both medial and lateral digits of the ungula [28] in the thoracic and pelvic limbs were separated and tagged. The skin and soft tissues were removed from the bones yielding the facies dorsalis and facies palmaris/plantaris of the bones. Seven different measurements for the facies medialis and lateralis of metacarpal and metatarsal bones were recorded as per Nacambo et al. [12]: Distance from the margo proximalis lateralis to the lateral (L1) and medial (D1) cartilago physialis; distance from the margo proximalis lateralis to the margo abaxialis of the lateral (L2) and medial (D2) caput; distance from cartilago physialis lateralis to the margo abaxialis of the lateral caput (X1); distance from the cartilago physialis medialis to the margo distalis of the caput ridge (X2); and distance from the margo axialis of the cartilago physialis axialis to the margo axialis of the lateral caput (X3) were recorded. All measurements were taken using a micrometer (722 Vernier Calliper; General Tools, Secaucus, NJ, USA) and a topographical representation of the measurements collected is presented in Fig. 3.

**Fig. 4:**
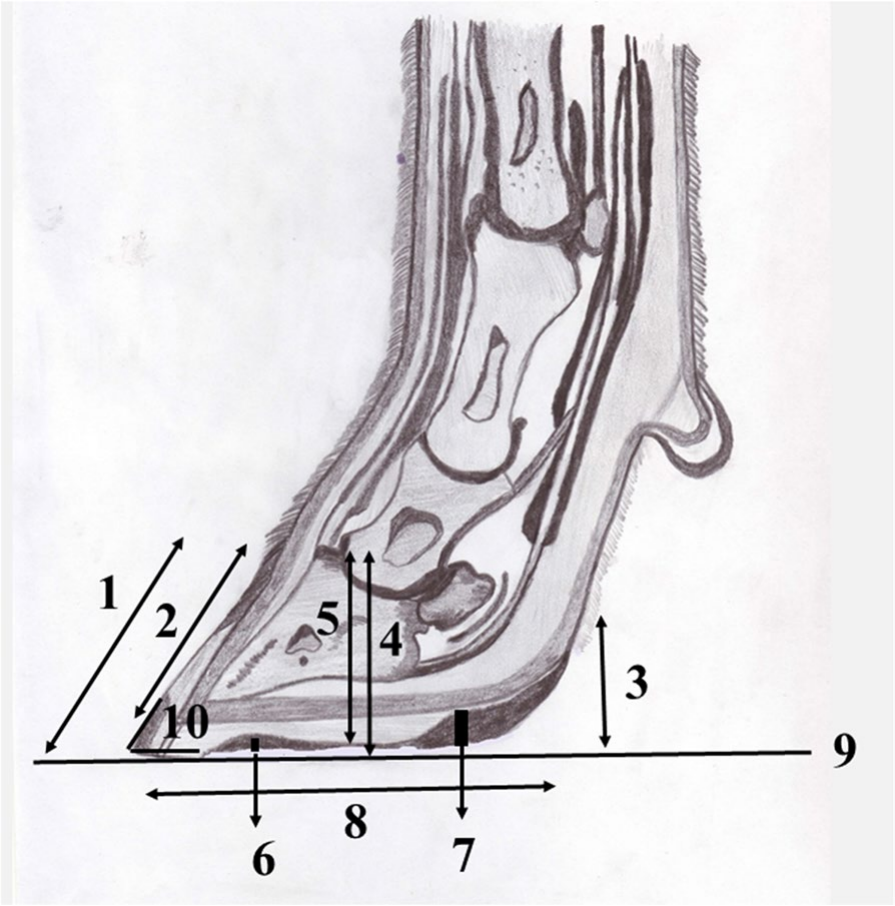
Topographical representation of different anatomical characteristics measured on thoracic and pelvic limbs of 2-year-old untrimmed pastured ewes from five different Iranian sheep breeds. **1)** Pars dorsalis length; **2)** length from the pars dorsalis to the corona; **3)** pars mobilis lateralis and medialis height; **4)** pars dorsalis height to ground; **5)** pars dorsalis height to the solea cornea; **6)** solea cornea thickness in the pars dorsalis region; **7)** solea cornea thickness in the pars mobilis lateralis and medialis region; **8)** solea length; **9)** Ground surface; **10)** pars dorsalis angle

Next, ungulae were cut sagittally and the following measurements were taken in each digit using a micrometer (722 Vernier Calliper; General Tools, Secaucus, NJ, USA) as per Mohamadnia et al. [29]: (i) pars dorsalis length, distance between the corona and the margo distalis of the pars dorsalis; (ii) pars dorsalis length to the corona, distance between the corona and the corium limbi; (iii) pars mobilis lateralis and medialis height, the distance between the plantar zona alba and the solea cornea at the regio pars mobilis lateralis and medialis; (iv) pars dorsalis height to the ground, the distance between the corona and the ground level at the pars dorsalis; (v) pars dorsalis height to the solea cornea, the distance between the corona and the solea cornea at the pars dorsalis region, (vi) solea cornea thickness in the pars dorsalis region; (vii) solea cornea thickness in the pars mobilis lateralis and medialis region, the thickness of the solea between the corium limbi and the solea cornea in the pars dorsalis, pars mobilis lateralis and medialis were considered as sole thickness; (viii) solea length, the distance between the of pars dorsalis distalis and the pars mobilis lateralis and medialis; and (ix) angulus dorsalis, the angle of the pars dorsalis was mathematically calculated from pars dorsalis height to solar surface and pars dorsalis length. Additionally, pars dorsalis height to solea cornea, to pars mobilis lateralis and medialis height ratio was calculated. A topographical representation of the measurements collected is presented in Fig. 4.

### Statistical analysis

All data were analysed in SAS v9.4 (SAS Inst. Inc., Cary, NC). Alpha level for determination of significance and trends were 0.05 and 0.10, respectively. Response variables were evaluated for normality using the Shapiro–Wilk test and by examining the normal plot. All response variables were normally distributed. To investigate breed differences in the length of metacarpal and metatarsal bones, data were analysed using mixed model equation methods using PROC MIXED. Models included breed, limbs (i.e. thoracic and pelvic), digit (i.e., lateral or medial), and their interactions as fixed effects. Multiple means comparisons were done using Tukey-Kramer’s correction. Results for fixed effects are reported as least square means ± standard error. To investigate the associations between length of bones and ungula measurements, Pearson’s correlations were calculated using PROC CORR.

## Supplementary Information

**Additional file 1: Supplementary Figure 1.** Measurements for anatomical characteristics of lateral and medial digits measured in the thoracic and pelvic limbs of 2-year-old untrimmed pastured ewes from five Iranian sheep breeds namely Afshari (AF), Kurdi (KD), Lori–Bakhtiari (LO), Moghani (MG), and Makoui (MK).

## Data Availability

The datasets used for the results presented in this study are available from the corresponding author upon reasonable request.
