# Supplementary figures and images for: Length of metacarpal and metatarsal bones in five Iranian sheep breeds and their associations with ungula measurements
Source: BMC Vet Res. 2021 Dec 6;17:376. doi: 10.1186/s12917-021-03076-5 (PMC8647440; doi:10.1186/s12917-021-03076-5)

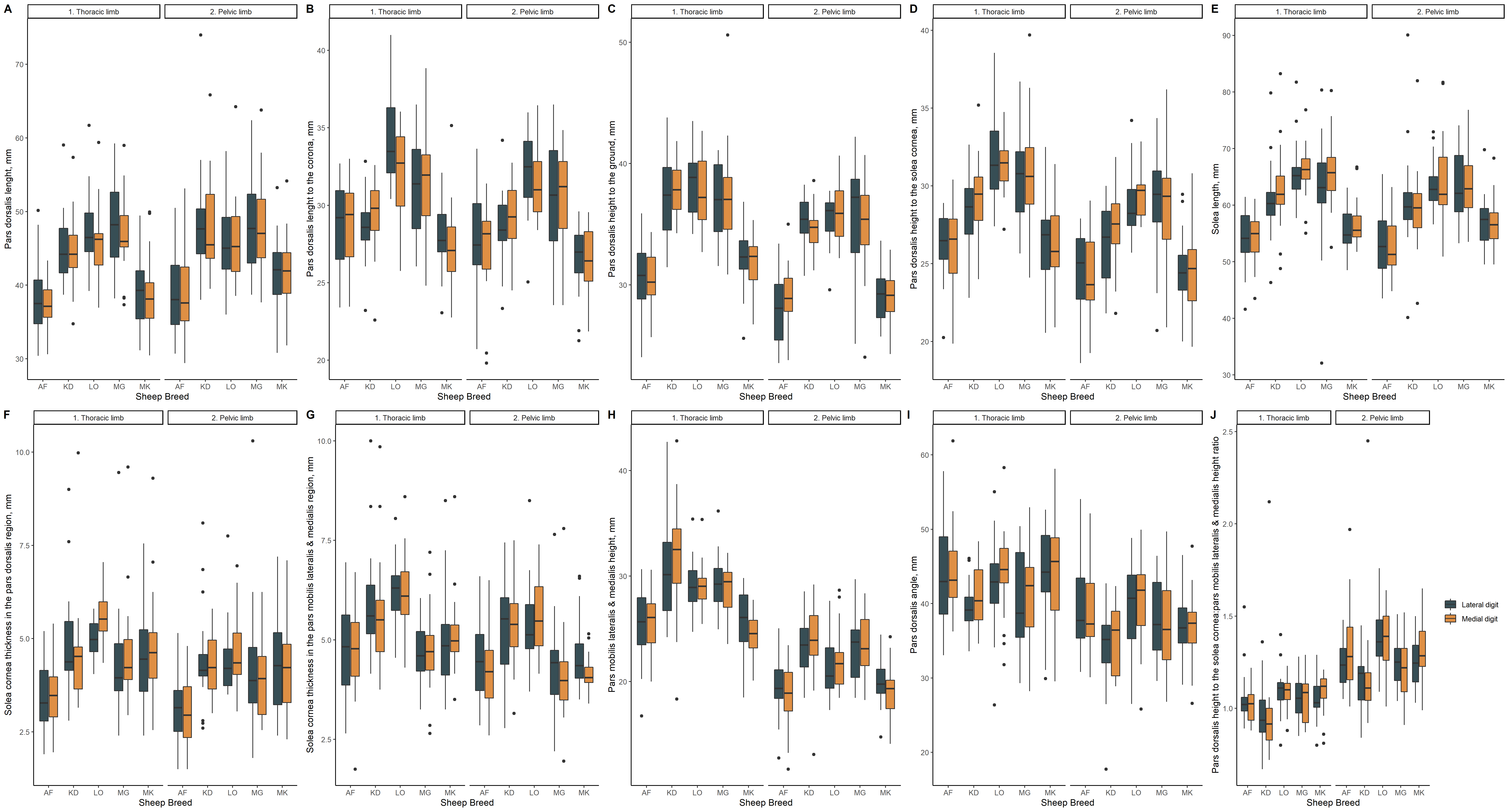

Supplement: Supplementary file 1 — Additional file 1: Supplementary Figure 1. Measurements for anatomical characteristics of lateral and medial digits measured in the thoracic and pelvic limbs of 2-year-old untrimmed pastured ewes from five Iranian sheep breeds namely Afshari (AF), Kurdi (KD), Lori–Bakhtiari (LO), Moghani (MG), and Makoui (MK). [file 12917_2021_3076_MOESM1_ESM.tiff]
